# Isolation of H5N6, H7N9 and H9N2 avian influenza A viruses from air sampled at live poultry markets in China, 2014 and 2015

**DOI:** 10.2807/1560-7917.ES.2016.21.35.30331

**Published:** 2016-09-01

**Authors:** Jie Zhou, Jie Wu, Xianqiao Zeng, Guofeng Huang, Lirong Zou, Yingchao Song, Divya Gopinath, Xin Zhang, Min Kang, Jinyan Lin, Benjamin J Cowling, William G. Lindsley, Changwen Ke, Joseph Sriyal Malik Peiris, Hui-Ling Yen

**Affiliations:** 1 School of Public Health, Li Ka Shing Faculty of Medicine, The University of Hong Kong, Hong Kong SAR, China; 2 These authors contributed equally to this work; 3 Guangdong Provincial Center for Disease Control and Prevention, Guangzhou, Guangdong, China; 4 Allergy and Clinical Immunology Branch, Health Effects Laboratory Division, National Institute for Occupational Safety and Health, West Virginia, United States

**Keywords:** avian influenza virus, modes of transmission, air sampling, live poultry markets

## Abstract

Zoonotic infections by avian influenza viruses occur at the human–poultry interface, but the modes of transmission have not been fully investigated. We assessed the potential for airborne and fomite transmission at live poultry markets in Guangzhou city and in Hong Kong Special Administrative Region (SAR), China, during 2014 and 2015. Viral genome and infectious avian influenza A viruses of H5N6, H7N9, and H9N2 subtypes were detected predominantly from particles larger or equal to 1 μm in diameter in the air sampled with cyclone-based bioaerosol samplers at the live poultry markets in Guangzhou. Influenza A(H9N2) viruses were ubiquitously isolated every month during the study period from air and environmental swabs, and different lineages of H9N2 virus were isolated from markets where chickens and minor land-based poultry were sold. The use of de-feathering devices increased the quantity of virus-laden airborne particles while market closure reduced the amount of such particles. The results highlight the possibility of airborne transmission of avian influenza viruses among poultry or from poultry to humans within such settings. This may explain epidemiological observations in which some patients with H7N9 infection reported being in markets but no direct contact with live poultry or poultry stalls.

## Introduction

Influenza A viruses infect a wide range of animal species and are transmitted via virus-laden particles through multiple non-exclusive modes. Interplay between multiple viral, host and environmental factors determine influenza viral transmission efficiency [1-5]. Virus–host compatibility establishes viral tropism and the quantity of virus-laden particles that may be released from infected hosts [1,2]. Gravity limits the distance that virus-laden particles can travel; large droplets settle rapidly and contribute to fomite transmission while droplet nuclei less than 5 μm in diameter may remain suspended in the air and mediate airborne transmission [3,4]. Humidity and temperature may impact on particle size and viability of the virus [5].

Zoonotic infections by avian influenza viruses occur at the human–avian interface [6] and live poultry markets play a critical role in maintaining, amplifying and disseminating avian influenza viruses between poultry species and from poultry to humans [7]. Exposure to live poultry has been reported by many patients with illness due to H5N1 and H7N9 infection, but sometimes such exposure has been indirect, for example visiting a vegetable stall within a large market where live poultry were sold [8]. Thus the modes of transmission are not well defined. The importance of contact or fomite transmission is supported by the detection of avian influenza viruses from various environmental swabs (e.g. counter surfaces, cages, water) at live poultry markets [9,10]. In addition, virus-laden particles that may mediate droplet or airborne transmission could be released from infected birds or as a result of aerosol-generating procedures during poultry slaughtering at markets. Currently, however, there is no information on the quantity, particle size and viability of virus-laden particles at live poultry markets.

To systematically assess the potential modes of transmission of avian influenza viruses at the human–poultry interface, we conducted monthly air and environmental sampling during July 2014 and October 2015 at three types of live poultry markets in Guangzhou city, Guangdong Province, China, and at one wholesale market in Hong Kong Special Administrative Region (SAR), China. In Hong King SAR, a ban on keeping live poultry overnight at retail live poultry markets has been implemented since 2008 [7]. 

## Methods

Samples were obtained from three different market types in Guangzhou: one wholesale market (two sites), one mixed animal market (two sites) and one retail market (one site). In Hong Kong SAR, we sampled at one wholesale poultry market. 

Sampling in the Guangzhou wholesale market and mixed animal market was carried out from July 2014 to October 2015. In the retail market, sampling was conducted from January to October 2015; in the Hong Kong SAR market, sampling was carried out in October and November in 2014 and March, April, July, August, September and October in 2015.

### Bioaerosol and environmental sampling at live poultry markets

Two types of cyclone-based bioaerosol samplers were used. The NIOSH bioaerosol sampler (BC251) collects particles based on their aerodynamic diameters into > 4, 1–4, and < 1 µm fractions at a flow rate of 3.5 L per minute [11]. The NIOSH samplers were set 1.2 m above ground and 0.5 m distance from poultry housing; samplers without connection to a vacuum pump were similarly placed as negative controls. After 30 min, a total of 0.105 m^3^ air was sampled; 1 mL of minimum essential media with 4% bovine serum albumin was added to each of the collection tubes and polytetrafluorethylene filters and transported on ice packs to the laboratories at Guangdong Provincial Center for Disease Control and Prevention or at the University of Hong Kong. 

The Coriolis μ air sampler (referred hereafter to as Coriolis) (Bertin Technologies) collects air at 100–300 L per minute. After 10 min sampling using 300 L per minute, a total of 3.0 m^3^ air was sampled into a conical vial containing 5 mL MEM, which was concentrated using the 100 kDa Amicon Ultra-15 (Millipore) to a final volume of 1.5 mL. The sampler was placed 1 m above the ground and 0.5 m distance from poultry housing. 

In parallel, environmental swabs were also collected from drinking water, fresh faecal droppings, or surfaces (cages, de-feathering machine and waste bins) at the markets. Temperature and humidity were recorded using a hygro-thermometer (Extech).

### Detection and quantification of influenza viral RNA genome 

Viral RNA for testing by quantitative real-time reverse transcription polymerase chain reaction (qRRT-PCR) was extracted from 400 µL of the specimen using the QIAGEN EZ Robot or the RNeasy Mini Kit (Qiagen) and eluted into 60 µL H_2_O. Influenza viral RNA was detected using AgPath-ID One-Step RT-PCR Reagents (Life Technologies) with specific primers and probes [12], using 5 µL of the eluted RNA. The number of influenza A virus M gene copies per m^3^ air was calculated, where V_w_ is the volume of medium added to the sampler, V_r_ is the volume of specimen used for RNA extraction, U is the airflow rate (m^3^ per minute) and t is the sampling time.

$$\text{M gene copies per cubic metre air }\text{= }\text{copies per }\text{µL}\;\text{× 60 µL × }\frac{{\text{V}}_{\text{w}}}{{\text{V}}_{\text{r}}}\text{ ÷ }\left(\text{U × t}\right)\;\;\;\;\;\;\;\;\;\;\;\;$$

The minimum linear range of quantification (LoQ) was two copies M gene per µL, and the LoQs were determined as 2,857 and 150 copies/m^3^ air for the NIOSH and Coriolis samplers, respectively. Influenza A virus M gene-positive samples were subtyped using H5-, H7- or H9-specific primers and probes by qRRT-PCR [9].

### Virus isolation in embryonated chicken eggs

All samples with threshold cycle (Ct) values ≤ 35 for influenza A virus M gene by qRRT-PCR were propagated in embryonated chicken eggs by injecting 0.2 mL of specimen into the allantoic cavity and incubated at 37 °C for 48–72 hours. Allantoic fluid that agglutinated chicken or turkey red blood cells were further characterised by qRRT-PCR; samples with increasing copy numbers for influenza viral H5, H7 or H9 gene (reduced Ct values relative to the original field samples) after egg propagation were considered positive by virus isolation.

### Genome sequencing and phylogenetic analysis

Viral RNA from an isolated virus was extracted using the RNeasy Mini Kit (Qiagen), amplified by RT-PCR [13] and was subjected to dideoxynucleotide sequencing or next-generation sequencing using the Ion PGM System with PathAmp FluA Reagents (Life Technologies). The sequences were submitted to the Global Initiative on Sharing All Influenza Data (GISAID) [14] (EPI674320, EPI674374 to EPI674424, EPI676397 to EPI676400, EPI676490, EPI676491, EPI696727 and EPI696728). Phylogenetic analysis was performed with the H9 haemagglutinin (HA) coding sequence (1,093 nt, 115–1,207 nt from ATG) aligned with reference strains from GISAID (Table 1). Phylogenetic trees were constructed by maximum likelihood method with bootstrap analysis (n = 1,000) by MEGA (version 6.0).

**Table 1:** Origin of the haemagglutinin sequences of influenza A(H9N2) isolates used for the phylogenetic analysis

Segment ID	Country	Collection date	Isolate name	Submitting laboratory	Authors
EPI573379	China	2014-Jul-04	A/chicken/Shanghai/015/2014	Shanghai Animal Disease Control Center	Ge,F., Mao,X. and Liu,J.
EPI339694	China	2011-Jul	A/chicken/Anhui/G29/2011	China Animal Health & Epidemiology Center	Chen,J., Liu,S., Jiang,W.M., Hou,G.Y., Li,J.P. and Chen,J.M.
EPI315926	China	2011-Feb	A/chicken/Anhui/ZTL/2011	South China Agricultural University	Liu,J.
EPI296439	China	2009-Jan	A/chicken/Baoshan/111/2009	Yunnan Tropical and Subtropical Animal Virus Diseases Laboratory	Song,J., Ye,C. and Tian,J.
EPI572786	China	2013-Mar-09	A/chicken/Beijing/0309/2013	China Agricultural University	Pu,J., Wang,J., Zhang,G., Yin,Y., Lv,N., Zhu,B., et al.
EPI470965	China	1994	A/chicken/Beijing/1/1994	St. Jude Children's Research Hospital	Baranovich,T., Marathe,B.M., Bridges,O., Burnham,A., Carey,D., Cline,T.D., et al.
EPI597003	China	2014-Feb-21	A/chicken/Dongguan/1424/2014	The University of Hong Kong	Lam,T.T., Zhou,B., Wang,J., Chai,Y., Shen,Y., Chen,X., et al.
EPI315960	China	2011-Jan	A/chicken/Fujian/SL6/2011	South China Agricultural University	Liu,J.
EPI81684	China	2000	A/chicken/Guangdong/4/00	Harbin Veterinary Research Institute	Li,C., Yu,K., Tian,G., Yu,D., Liu,L., Jing,B., et al.
EPI315932	China	2011-Feb	A/chicken/Guangdong/FZH/2011	South China Agricultural University	Liu,J.
EPI621363	China	2015-Jan	A/chicken/Guangdong/KPL01/2015	South China Agricultural University	Su,X. and Xie,Q.
EPI239451	China	1994	A/Chicken/Guangdong/SS/94	Yangzhou University	Shi,H.Y., Liu,X.F., Chen,S.J., Sun,L. and Xin,C.H.A.
EPI81694	China	1999	A/chicken/Guangxi/10/99	Harbin Veterinary Research Institute	Li,C., Yu,K., Tian,G., Yu,D., Liu,L., Jing,B., et al.
EPI122456	China	2005	A/chicken/Guangxi/55/2005	Guangxi General Veterinary Prevention and Quarantine Services	Liu,Q., Xiong,Y., Qin,L., Liu,K., Zhu,W., Qin,F.Y., et al.
EPI122438	China	2000	A/chicken/Guangxi/6/2000	Guangxi General Veterinary Prevention and Quarantine Services	Liu,Q., Xiong,Y., Qin,L., Liu,K., Zhu,W., Qin,F.Y., et al.
EPI538912	China	2013-Nov-23	A/chicken/Guangxi/LS/2013	Guangxi Veterinary Research Institute	Li,H.M., Guo,J.G., Zhou,L.B., Chen,L., Long,J.M. and Pan,J.
EPI81698	China	2000	A/chicken/Hebei/31/00	Harbin Veterinary Research Institute	Li,C., Yu,K., Tian,G., Yu,D., Liu,L., Jing,B., et al.
EPI140897	China	2008-Aug-02	A/chicken/Hebei/B1/2001	Shandong Academy of Agricultural Science, Animal Husbandry and Veterinary Institute	Huang,Y., Hu,B., Wen,X., Cao,S., Gavrilov,B.K., Du,Q., et al.
EPI140900	China	2006	A/chicken/Hebei/L1/2006	Shandong Animal Husbandry and Veterinary Institute	Huang,Y., Hu,B., Wen,X., Cao,S., Xu,D., Zhang,X., et al.
EPI254331	China	2003	A/chicken/Heibei/8/2003	College of Life Science and Technology, Southwest University of Nationalities	Yue,H., Tang,C. and Li,M.Y.
EPI326397	China	1998	A/chicken/Henan/A3/1998	Henan Agriculture University	Wang,Z., Zhao,J., Cai,L., Zheng,L. and Wang,C.
EPI238802	China	1998	A/chicken/Henan/nd/1998	Beijing Genomics Institute, Chinese Academy of Sciences	Liao,X., Zhang,X., Wang,J., Yu,J. and Liu,J.
EPI470859	Hong Kong SAR	1997	A/chicken/Hong_Kong/G9/1997	St. Jude Children's Research Hospital	Baranovich,T., Marathe,B.M., Bridges,O., Burnham,A., Carey,D., Cline,T.D., et al.
EPI470915	Hong Kong SAR	2011	A/chicken/Hong_Kong/NT10/2011	St. Jude Children's Research Hospital	Baranovich,T., Marathe,B.M., Bridges,O., Burnham,A., Carey,D., Cline,T.D., et al.
EPI337045	China	1999-Feb-21	A/chicken/Hubei/01/1999	Huazhong Agricultural University	Zhang,Z., Hu,S., Li,Z., Liu,M., Li,S., Xiao,Y. et al.
EPI623618	China	2014-May-28	A/chicken/Hubei/2014	Wuhan Institute of Virology, Chinese Academy of Sciences	Wang,N., Liu,X.-J., Wang,B., Zhang,S.-H. and Ge,X.-Y.
EPI593620	China	2014-Jun-29	A/chicken/Jiangxi/19426/2014	Centre of Influenza Research, School of Public Health, The University of Hong Kong	Lam,T.T., Zhou,B., Wang,J., Chai,Y., Shen,Y., Chen,X., et al.
EPI559917	China	2012-Nov-10	A/chicken/Jilin/GYH1/2012	College of Veterinary Medicine, Jilin University	Cong,Y., Zhu,L. and Ran,W.
EPI301458	Jordan	2004	A/chicken/Jordan/1436-1451/2004	Istituto Zooprofilattico Sperimentale Delle Venezie	Fusaro,A., Monne,I., Salviato,A., Valastro,V., Schivo, A., Amarin,N.M., et al.
EPI470818	South Korea	1996	A/chicken/Korea/25232-96006/1996	St. Jude Children's Research Hospital	Baranovich,T., Marathe,B.M., Bridges,O., Burnham,A., Carey,D., Cline,T.D., et al.
EPI5901	South Korea	1996	A/Chicken/Korea/38349-p96323/96	St. Jude Children's Research Hospital	Guan,Y., Shortridge,K.F., Senne,D., Krauss,S. and Webster,R.G.
EPI304611	South Korea	2007-Nov-24	A/chicken/Korea/GH2/2007	Biological Sciences, Inje University	Koo,Y.
EPI339649	China	2011-Jul	A/chicken/Ningxia/182/2011	Laboratory of Animal Epidemiological Surveillance, China Animal Health & Epidemiology Center	Chen,J., Liu,S., Jiang,W.M., Hou,G.Y., Li,J.P. and Chen,J.M.
EPI487143	China	2001-Jul-18	A/chicken/Shandong/241/2001	Avian Virus Disease Laboratory, LanZhou Veterinary Research Institute, Chinese Academy of Agricultural Sciences	In,Z., Xu,C., Liu,B., Ji,Y., Fu,Y., Guo,J. and et al.
AF508570**^a^**	China	1996	A/Chicken/Shandong/6/96	Microbiology, The University of Hong Kong	Li,J.W., Yu,K.Z., Brown,I., Shortridge,K.F., Pieris,J.S.M. and Guan,Y.
EPI470818	China	2009-May	A/chicken/Shandong/H/2009	CAS Key Laboratory of Pathogenic Microbiology and Immunology, Institute of Microbiology, Chinese Academy of Sciences	Lu,L., Bi,Y., Li,J., Sun,L. and Liu,W.
EPI272334	China	2008-Dec-01	A/chicken/Shandong/LY/2008	Department of Veterinary Prevention Medicine, College of Veterinary Medicine, China Agricultural University	Pu,J., Zhang,G. and Liu,J.
EPI239002	China	1998	A/Chicken/Shanghai/F/98	Animal Infectious Disease Laboratory, School of Veterinary Medicine, Yangzhou University	Lu,J.H., Liu,X.F., Shao,W.X., Liu,Y.L., Wei,D.P. and Liu,H.Q.
EPI470907	China	2005	A/chicken/Shantou/22116/2005	St. Jude Children's Research Hospital	Baranovich,T., Marathe,B.M., Bridges,O., Burnham,A., Carey,D., Cline,T.D., et al.
AF508572**^a^**	China	1997	A/chicken/Shenzhen/9/97	Microbiology, The University of Hong Kong	Li,J.W., Yu,K.Z., Brown,I., Shortridge,K.F., Pieris,J.S.M. and Guan,Y.
EPI573766	China	2014-Mar	A/chicken/Yangzhou/752/2014	Yang Zhou University	Yang,X.
EPI241325	China	2000-Sep-04	A/chicken/Zhejiang/HE6/2009	College of Animal Science, South China Agricultural University	Chen,C., Ji,J., Bai,S., Zuo,K. and Xie,Q.
EPI221855	China	2007	A/chicken/Zhejiang/HJ/2007	Avian Disease, Animal Husbandry and Veterinary Medicine of Fujian Academy of Agricultural Sciences	Wan,C. and Huang,Y.
EPI610285	China	2010-Oct-10	A/Chinese francolin/Guangxi/B7/2010	Guangxi Key Laboratory of Animal Vaccines and Diagnostics, Guangxi Veterinary Research Institute	Peng,Y., Xie,Z., Liu,J., Pang,Y., Xie,Z., Xie,L., et al.
EPI441826	Viet Nam	2006-Dec	A/Chinese Hwamei/Vietnam/38/2006	Kyoto Sangyo University, Faculty of Life Sciences	Takakuwa,H.
CY005632**^a^**	Hong Kong SAR	1979-Oct-09	A/duck/HK/784/1979	St. Jude Children's Research Hospital	Obenauer,J.C., Denson,J., Mehta,P.K., Su,X., Mukatira,S., Finkelstein,D.B., et al.
EPI10783	Hong Kong SAR	2003	A/Duck/Hong_Kong/289/78	Microbiology, The University of Hong Kong,	Li,K.S., Xu,K.M., Peiris,J.S., Poon,L.L., Yu,K.Z., Yuen,K.Y., et al.
EPI16562	Hong Kong SAR	1979	A/duck/Hong_Kong/552/79	Microbiology, The University of Hong Kong	Li,K.S., Xu,K.M., Peiris,J.S., Poon,L.L., Yu,K.Z., Yuen,K.Y., et al.
EPI5885	Hong Kong SAR	1997	A/Duck/Hong_Kong/Y280/97	St. Jude Children's Research Hospital	Guan,Y., Shortridge,K.F., Krauss,S. and Webster,R.G.
EPI5887	Hong Kong SAR	1997	A/duck/Hong_Kong/Y439/1997	St. Jude Children's Research Hospital	Baranovich,T., Marathe,B.M., Bridges,O., Burnham,A., Carey,D., Cline,T.D., et al.
EPI497495	China	2013-Mar-13	A/environment/Chongqing/00516/2013	WHO Chinese National Influenza Center	Gao, R., Li, X., Zhang, Y., Zou, S., Zhao, X., Li, X., et al.
EPI457842	China		A/environment/Jiangxi/02895/2012	Harbin Veterinary Research Institute	Li,C., Yu,K., Tian,G., Yu,D., Liu,L., Jing,B., and et al.
EPI492254	China	2013-Mar-25	A/environment/Zhejiang/14/2013	Zhejiang Provincial Center for Disease Control and Prevention	Feng,Y., Mao,H., Xu,C., Jiang,J., Chen,Y., Yan,J., et al.
EPI232381	USA	2008	A/ferret/Maryland/P10-UMD/2008	University of Maryland	Sorrell,E.M., Wan,H., Araya,Y., Song,H. and Perez,D.R.
AJ404627**^a^**	Hong Kong SAR	1999	A/guinea_fowl/Hong_Kong/WF10/99	St. Jude Children's Research Hospital	Perez,D.R., Lim,W., Seiler,J.P., Yi,G., Peiris,M., Shortridge,K.F. et al.
EPI24300	Hong Kong SAR	2003	A/guineafowl/HongKong/NT184/03	St. Jude Children's Research Hospital	Choi,Y.K., Ozaki,H., Webby,R.J., Webster,R.G., Peiris,J.S., Poon,L., et al.
AJ404627**^a^**	Hong Kong SAR	1999	A/Hong_Kong/1074/99	Virology, National Institute for Medical Research, London	Lin,Y.P., Shaw,M., Gregory,V., Cameron,K., Lim,W., Klimov,A., et al.
EPI498037	Hong Kong SAR	2013-Dec-28	A/Hong_Kong/308/2014	Public Health Laboratory Services Branch, Centre for Health Protection	Mak,G.C., Cheng,P.K.C., Lo,J.Y.C.
CY055140**^a^**	Hong Kong SAR	2009-Oct-29	A/Hong_Kong/33982/2009	Public Health Lab Centre, Centre For Health Protection, Department of Health, Hong Kong	Cheng,P.K.C. and Lim,W.L.
EPI439653	China	2011-Feb	A/mallard/Jiangxi/42/2011	Institute of Molecular Ecology and Evolution, East China Normal University	Zhu,G., Wang,R., Xuan,F., Daszak,P., Anthony,S.J., Zhang,S., Zhang,L. and He,G.
EPI5889	Hong Kong SAR	1997	A/Quail/Hong_Kong/G1/97	St. Jude Children's Research Hospital	Guan,Y., Shortridge,K.F., Krauss,S. and Webster,R.G.
EPI573570	China	2013-Apr	A/quail/Jiangsu/WX3/2013	School of Veterinary Medicine, Yangzhou University	Zhu,Y., Yang,D., Ren,Q., Yang,Y., Liu,X., Xu,X., et al.
EPI113630	China	2005	A/quail/Shantou/19506/2005	Department of Microbiology, The University of Hong Kong,	Xu,K.M., Li,K.S., Smith,G.J.D., Li,J.W., Tai,H., Zhang,J.X., et al.
EPI113492	China	2000	A/quail/Shantou/782/2000	Department of Microbiology, The University of Hong Kong,	Xu,K.M., Li,K.S., Smith,G.J.D., Li,J.W., Tai,H., Zhang,J.X., et al.
EPI597411	China	2014-Feb-21	A/silkie_chicken/Dongguan/968/2014	Centre of Influenza Research, School of Public Health, The University of Hong Kong	Lam,T.T., Zhou,B., Wang,J., Chai,Y., Shen,Y., Chen,X., et al.
EPI5913	United States	1966	A/turkey/California/189/66	St. Jude Children's Research Hospital	Guan,Y., Shortridge,K.F., Krauss,S. and Webster,R.G.
EPI407924	United States	1966	A/turkey/Wisconsin/1/1966	St. Jude Children's Research Hospital	Wentworth,D.E., Dugan,V., Halpin,R., Lin,X., Wester,E., Bera,J., et al.

^a^ Sequence downloaded from Influenza Research Database funded by the National Institute of Allergy and Infectious Diseases (NIAID) [36].

### Statistical analysis

Correlation analyses were done by determining Spearman’s rank-correlation coefficients (r_s_). Fisher’s exact test was applied to assess if the subtypes detected were statistically significantly different. Statistical analyses were performed using Graphpad Prism 6.0.

### Ethics statement

Permission from the vendors at the poultry markets was obtained before the bioaerosol and environmental sampling. All sampling was performed without directly handling the poultry, thus animal ethics were not applicable for our study.

## Results

### Sampling at a wholesale market in Guangzhou

The wholesale market was organised into areas for holding live poultry, slaughtering and selling dressed poultry (poultry carcasses). Two sites were sampled. Site A1 was within the live poultry holding area of ca 5,500 m^2^, where 10,000–20,000 poultry (predominantly chickens) were kept at any one time. Chickens were kept on a litter-bedded floor and were often sold to other retail markets within three days. Site A2 was a stall for chicken slaughtering with a de-feathering machine. There was one routinely scheduled market rest day per month; additional rest days may be scheduled in response to reports of human zoonotic infections.

#### Site A1

**Figure 1:**
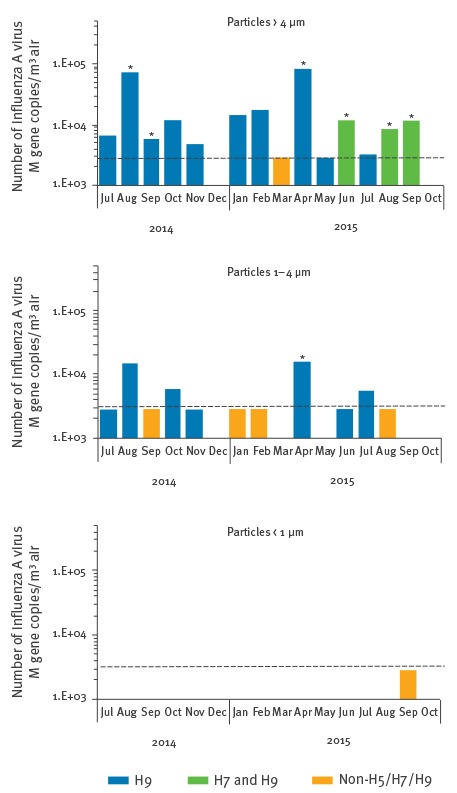
Influenza A virus M gene copy number from particles in air sampled at a wholesale live poultry market in Guangzhou city, China, July 2014–October 2015

Using the NIOSH sampler, influenza A virus M gene was detected by qRRT-PCR from particles > 4 µm in 14/16 samples at 3,300–79,357 copies/m^3^ air, with 2/14 samples positive for the M gene but below the LoQ. In addition, the M gene was detected from particles 1–4 µm in 11/16 samples at 5,578–15,536 copies/m^3^ air (7/11 below LoQ) and from particles < 1 µm (1/16 sampling, 1/1 below LoQ) (Figure 1). In parallel, NIOSH samplers without a connection to a vacuum pump (as negative controls) were consistently negative for influenza A virus M gene by qRRT-PCR from particles > 4, 1–4 or < 1 µm. H9 was the predominant HA subtype detected by qRRT-PCR, while mixed H7 and H9 or non-H5/H7/H9 RNA were also detected (Figure 1, Table 2).

The quantity and subtypes of influenza virus-laden particles detected in the air using a NIOSH bioaerosol sampler, at particle sizes of > 4, 1–4 and < 1 μm diameter are shown. The horizontal dotted lines indicate the linear range of quantification for the influenza A virus M gene by quantitative real-time reverse transcription polymerase chain reaction (qRRT-PCR) assay. Samples in which virus was isolated after egg passage are indicated by an asterisk (*).

**Table 2:** Influenza A viruses detected and isolated from air and environmental samples at live poultry markets, Guangzhou, China (3 markets), and Hong Kong SAR (1 market), July 2014–October 2015^a^

Market and sample type	Number of influenza A virus M gene-positive^b^/ total sampled	Number of isolates/number of influenza A virus M gene-positive samples^c^	HA subtype of influenza A virus M gene-positive samples^d^ (number of isolates)						
			H5	H7	H9	H5 and H9	H7 and H9	H5 and H7	Non-H5/H7/H9
**Wholesale market, Guangzhou** ^e^									
Air (NIOSH sampler)									
Particles > 4 µm	14/16	6/14	0	0	10 (3)	0	3 (3)	0	1
Particles 1–4 µm	11/16	1/11	0	0	7 (1)	0	0	0	4
Particles < 1 µm	1/16	0/1	0	0	0	0	0	0	1
Air (Coriolis μ)	12/14	4/12	0	0	9 (3)	0	3 (1)	0	0
Drinking water	8/11	4/8	0	0	3 (1)	0	5 (3)	0	0
Faecal droppings and surfaces	28/48	11/28	0	0	16 (9)	0	9 (2)	0	3
**Mixed animal market, Guangzhou^f^**									
Air (NIOSH sampler, site B1)									
Particles > 4 µm	15/16	5/15	0	0	11 (3)	2 (2)	0	0	2
Particles 1–4 µm	9/16	0/9	0	0	7	0	0	0	2
Particles < 1 µm	1/16	0/1	0	0	0	0	0	0	1
Air (NIOSH sampler, site B2)									
Particles > 4 µm	15/16	4/15	0	0	12 (2)	2 (2)	0	0	1
Particles 1–4 µm	11/16	0/11	0	0	7	0	0	0	4
Particles < 1 µm	3/16	0/3	0	0	0	0	0	0	3
Air (Coriolis μ)	14/14	6/14	0	0	10 (4)	3 (2)	0	0	1
Drinking water	11/30	3/11	1 (1)	1	5 (2)	1	0	1	2
Faecal droppings and surfaces	54/79	15/54	4 (2)	1	32 (12)	3	1	2	11 (1)
**Retail market, Guangzhou**									
Air (NIOSH sampler)									
Particles > 4 µm	10/10	1/10	0	0	5 (1)	2	1	0	2
Particles 1–4 µm	6/10	0/6	0	0	2	0	0	0	4
Particles < 1 µm	1/10	0/1	0	0	0	0	0	0	1
Drinking water	4/13	0/4	0	1	1	0	1	0	1
Faecal droppings and surfaces	14/23	1/14	0	1	11 (1)	0	1	0	1
**Wholesale market, Hong Kong SAR^g^**									
Air (NIOSH sampler)	0/22	0/0	0	0	0	0	0	0	0
Air (Coriolis μ)	6/13	0/6	0	0	3	0	0	0	3
Faecal droppings and surfaces	0/39	0/0	0	0	0	0	0	0	0

HA: haemagglutinin; qRRT-PCR: quantitative real-time reverse transcription polymerase chain reaction.

^a^ In the retail market in Guangzhou, sampling was conducted from January to October 2015; in the market in Hong Kong SAR, sampling was conducted in October and November in 2014 and in March, April, July, August, September and October in 2015.

^b^ Influenza A virus M gene was detected using qRRT-PCR.

^c^ The virus isolation rate was defined as the number of positive isolates after one passage in embryonic chicken eggs among influenza A virus M gene-positive samples.

^d^ The M gene-positive samples were further subtyped by qRRT-PCR using primers and probes for H5, H7, H9 HA.

^e^ The sampling site was located at the poultry holding area within the wholesale live poultry market (see site A1 in the text).

^f^ Sites B1 and B2 were two separate vendors’ stalls within the mixed animal market.

^g^ No drinking water was provided in the wholesale market in Hong Kong SAR.

H9N2 viruses (five isolates) and mixture of H7N9/H9N2 viruses (one isolate) were further isolated from the air samples collected by the NIOSH sampler at the fraction of > 4 μm, with an isolation rate (number of isolates/number of PCR-positive samples) of 6/14 (Tables 2 and 3). From the fraction of 1–4 μm, one H9N2 virus was isolated from 11 influenza A virus M gene-positive samples after egg propagation (Table 2).

**Table 3:** Influenza A virus isolation from samples with mixed H5, H7, H9 haemagglutinin subtypes from two live poultry markets in Guangzhou, China, July 2014–October 2015

Sample type	Sample ID	Date	HA subtype(s) detected		
			In market samples by qRRT-PCR		After egg passage^a^
**Wholesale market, Guangzhou^b^**					
NIOSH air sample	GZ331	Jun 2015	H7 and H9		H9
	GZ395	Aug 2015	H7 and H9		H7 and H9
	GZ437	Sep 2015	H7 and H9		H9
Coriolis μ air sample	GZ449	Sep 2015	H7 and H9		H9
Drinking water	GZ376	Aug 2015	H7 and H9		H7 and H9
	GZ378	Aug 2015	H7 and H9		H7 and H9
	GZ417	Sep 2015	H7 and H9		H9
Faecal droppings	GZ319	Jun 2015	H7 and H9		H7 and H9
	GZ420	Sep 2015	H7 and H9		H9
**Mixed animal market, Guangzhou^c^**					
NIOSH air sample (site B1)	GZ089	Oct 2014	H5 and H9	H5 and H9	
	GZ184	Jan 2015	H5 and H9	H5	
NIOSH air sample (site B2)	GZ124	Nov 2014	H5 and H9	H5 and H9	
	GZ187	Jan 2015	H5 and H9	H5 and H9	
Coriolis μ air sample (both sites B1 and B2)	GZ259	Mar 2015	H5 and H9	H9	
	GZ289	Apr 2015	H5 and H9	H9	

HA: haemagglutinin; qRRT-PCR: quantitative real-time reverse transcription polymerase chain reaction.

^a^ A sample with copy numbers of influenza A virus H5, H7, or H9 genes (reduced threshold cycle (Ct) values by qRRT-PCR) higher than those of the original filed sample after egg propagation was considered positive by virus isolation.

^b^ The sampling site was located at the poultry holding area within the wholesale live poultry market (see site A1 in the text).

^c^ Sites B1 and B2 were two separate vendors’ stalls within the mixed animal market.

The HA and neuraminidase (NA) genes of the sample with mixed H7N9 and H9N2 (A/Environment-air/GZ/NIOSH-395/2015) from our study showed 99.3% and 99.6% homology to that of the A/Chicken/Guangdong/GZ068/15 (H7N9) virus (GISAID:EPI_ISL_176834), respectively. 

The Coriolis air sampler showed comparable efficiency to the NIOSH sampler in detecting influenza A virus M gene in the air samples, with Spearman’s r_s_ = 0.68 (p = 0.01). Influenza A virus M gene was detected from 12 of 14 samples at 310–21,413 copies/m^3^ air (Table 2). Four H9N2 viruses were isolated after one passage in embryonated eggs from 12 influenza A virus M gene-positive Coriolis samples, including one that was originally positive for both H9 and H7 RNA by qRRT-PCR (Tables 2 and 3).

Influenza A virus M gene was detected in 36 of 59 environmental swabs – with a total isolation rate of 15/36 – including drinking water, faecal droppings and surfaces (Table 2). Of samples that were influenza A virus M gene-positive, further subtyping demonstrated the H9 subtype (19/36), mixed H7/H9 (14/36) and non-H5/H7/H9 specimens (3/36). A total of 12 H9N2 viruses and three mixtures of H7N9/H9N2 viruses were isolated (Table 2 and 3). The distribution of virus subtypes detected in the environmental swabs and the NIOSH air samplers were not significantly different (p = 0.51, Fisher’s exact test).

We analysed if viral load or environmental conditions might be associated with virus isolation from the air samplers; however, the M gene copy numbers, temperature, and relative humidity were not significantly different between months in which virus was isolated and those in which it was not, using the NIOSH sampler (p = 0.17, 0.07 and 0.72, respectively, Mann–Whitney test) or the Coriolis sampler (p = 0.86, 0.49 and 0.32, respectively). 

In December 2014 and October 2015, neither air sampler detected the influenza A virus M gene. In December 2014, sampling was coincidentally performed on the market rest day (when the market was closed); all chickens were removed from the market but the environment had not yet been disinfected. In October 2015, sampling was performed the day after market closure. These results suggest that market closure may effectively reduce the viral load at the markets for a short time period.

#### Site A2

We performed air sampling while the de-feathering machine at site A2 was in operation (five samples) or not in use (three samples). While the machine was in operation, influenza A virus M gene was detected by qRRT-PCR from particles > 4 µm in 5/5 samples at 4,157–28,929 copies/ m^3^ air (2/5 below LoQ) and from particles 1–4 µm in 2/5 samples (2/2 below LoQ); no viral RNA was detected from particles < 1 µm (0/5 samples). H9 RNA was detected in 4/5 samples and mixed H5/H9 RNA was detected in 1/5 samples from particles > 4 µm; one H9N2 virus was isolated from the air sample.

In contrast, influenza A virus M gene was not detected in air sampled while the de-feathering machine was not in use (0/3 samples). At the same time, environmental swabs collected from the de-feathering machine were consistently positive for the M gene by qRRT-PCR, regardless of whether the machine was in use or not. Overall, the results suggest that infectious influenza A virus-laden particles can be generated during the de-feathering process.

### Sampling at a mixed animal market in Guangzhou

This mixed animal market sold live poultry, reptiles and mammals, although poultry were kept in a separate area. The predominant poultry species sold were aquatic birds (ducks and geese) and minor land-based poultries (pheasants, guinea fowls, chukar partridges, quails). Each vendor may have a few hundred birds of different species, which were kept in separate cages or pens of various sizes. There was no clear all-in/all-out policy or known routine market rest days.

**Figure 2:**
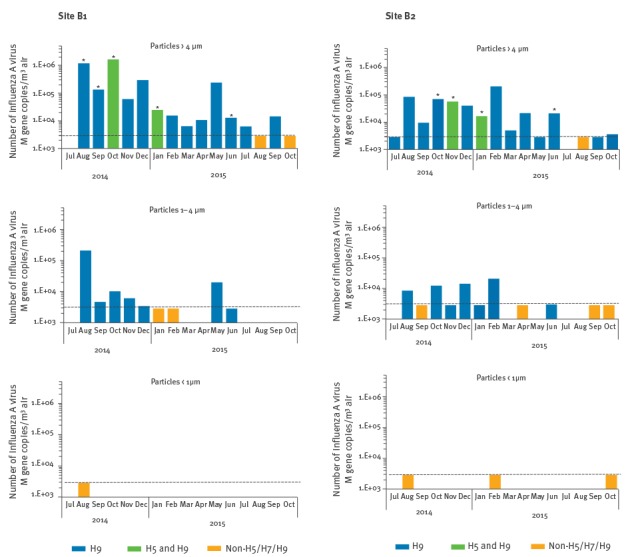
Influenza A virus M gene copy number from particles in air sampled at two separate vendors in a mixed animal market in Guangzhou city, China, July 2014–October 2015

NIOSH samplers were set up at two separate vendors’ stalls (sites B1 and B2). At site B1, influenza A virus M gene was detected by qRRT-PCR from particles > 4 µm in 15/16 samples at 6,179–1,650,000 copies/m^3^ air (2/15 below LoQ), from particles 1–4 µm in 9/16 samples at 3,450–210,714 copies/m^3^ air (3/9 below LoQ) and from particles < 1 µm in 1/16 samples (1/1 below LoQ) (Figure 2). At site B2, influenza A virus M gene was detected from particles > 4 µm in 15/16 samples at 3,590–204,286 copies/m^3^ air (4/15 below LoQ), from particles 1–4 µm in 11/16 samples at 3,050–20,857 copies/m^3^ air (6/11 below LoQ) and from particles < 1 µm in 3/16 sampling (3/3 below LoQ) (Figure 2).

The quantity and subtypes of influenza virus-laden particles at particle sizes at > 4, 1–4 and < 1 μm detected in the air using a NIOSH bioaerosol sampler at two sampling sites (sites B1 and B2) within the market are shown. The horizontal dotted lines indicate the linear range of quantification for influenza A virus M gene by quantitative real-time reverse transcription polymerase chain reaction (qRRT-PCR) assay. Samples in which virus was isolated after one passage in embryonated eggs are indicated by an asterisk (*).

H9 and mixed H5/H9 RNA were detected from the M gene-positive samples by qRRT-PCR. H9N2 (n = 6) and mixed H9N2/H5N6 (n = 3) viruses were isolated from the fraction of particles > 4 µm, with isolation rates of 5/15 and 4/15 at sites B1 and B2, respectively (Tables 2 and 3). Higher M gene copy numbers (p = 0.01, Mann–Whitney test) and lower relative humidity (p = 0.04) were noted in the months when influenza virus was isolated in air sampled by the NIOSH sampler. Using the Coriolis sampler, influenza A virus M gene was detected from 14/14 samples at 201–29,888 copies/m^3^ air (1/14 below LoQ), which were subsequently confirmed as H9 or mixed H5/H9 subtypes. Six H9N2 viruses were isolated from 14 air samples collected by the Coriolis sampler (Table 2).

Influenza A viral RNA was detected from 60% (65/109) environmental swabs (water, faecal droppings and surfaces), with an isolation rate of 18/65. H9 (37/65), H5 (5/65), H7 (2/65), mixed H5/H9 (4/65), mixed H5/H7 (3/65), mixed H7/H9 (1/65) or non-H5/H7/H9 (13/65) were further identified by qRRT-PCR (Table 2). H9N2 (n = 14), H5N6 (n = 2), H5N2 (n = 1), and H4N8 (n = 1) viruses were isolated. The subtypes detected in samples obtained using the NIOSH bioaerosol sampler and in environmental swabs were not statistically different (p = 0.27, Fisher’s exact test). Overall, five H5N6 viruses (three mixed with H9N2) and one H5N2 virus were isolated from the air and environmental samples. The H5 isolates belonged to clade 2.3.4.4 with 94.0–99.0% homology to the human H5N6 virus A/Guangzhou/39715/2014 (GISAID: EPI_ISL_180669) [15]. 

### Sampling at a retail market in Guangzhou

This retail market had 10 stalls that sold live poultry. Sampling was performed in one stall of 4 m^2^, which held 30–50 birds daily (co-housed chickens, ducks, pigeons, geese and quails). There were no clear all-in/all-out policy or known regular market rest days for disinfection. Sampling at the retail market was conducted from January to October 2015.

Using the NIOSH sampler, influenza A virus M gene was detected by qRRT-PCR from particles > 4 µm in 10/10 samples at 9,243–455,714 copies/m^3^ air (6/10 below LoQ), particles 1–4 µm in 6/10 samples at 3,130–14,071 copies/m^3^ air (4/6 below LoQ) and particles < 1 µm (1/10 samples, 1/1 below LoQ). H9 RNA was predominantly detected while mixed H7/H9 and H5/H9 RNA were also detected by qRRT-PCR. One H9N2 virus was isolated from particles > 4 µm among 10 samples positive for influenza A virus M gene.

The viral M gene was detected in 18/36 environmental swabs from drinking water, faecal droppings and surfaces; further subtyping identified H9 RNA (12/18), H7 RNA (2/18), mixed H7/H9 RNA (2/18) and non-H5/H7/H9 RNA (2/18), with one H9N2 virus isolated (Table 2). The subtypes detected by qRRT-PCR from the environmental swabs were not significantly different from those detected in the air samples obtained using the NIOSH sampler (p = 0.45, Fisher’s exact test).

### Sampling at a wholesale poultry market in Hong Kong SAR

This wholesale poultry market served as a temporary holding site for chickens imported from mainland China or raised locally. The chickens stayed for no longer than 48 hours until sold to retail markets, with a first-in/ first-out policy, segregation and strict biosecurity measures. Since 2013, chickens imported from mainland China and those raised locally have been housed separately at different locations.

Sampling was conducted in the area holding local poultry in October and November in 2014 as well as in March, April, July, August, September and October in 2015. At each sampling, NIOSH (n = 2–3) and Coriolis (n = 1–2) samplers were set up and there were varying numbers of chickens (between 50 and 500) in the holding area. Influenza A virus M gene was not detected by qRRT-PCR in any of the 22 NIOSH samples but was detected in 6/13 Coriolis samples at 203–470 copies/m^3^ air (3/6 below LoQ). Further subtyping identified H9 (3/6) or non-H5/H7/H9 (3/6) RNA from the M gene-positive samples (Table 2). Furthermore, none of the 39 environmental swabs were positive for the influenza A virus M gene (Table 2). The quantity of influenza A virus-laden particles in the air by the Coriolis sampler at this wholesale live poultry market in Hong Kong SAR (203–470 copies/m^3^, M gene-positive rate: 6/13, 3/6 below LoQ) was lower than that for the wholesale live poultry market (310–21,413 copies/m^3^, M gene-positive rate: 12/14) or the mixed animal market (201–29,888 copies/m^3^, M gene-positive rate: 14/14, 1/14 below LoQ) in Guangzhou city.

### Genetic analysis of H9N2 viruses isolated from the live poultry markets

**Figure 3:**
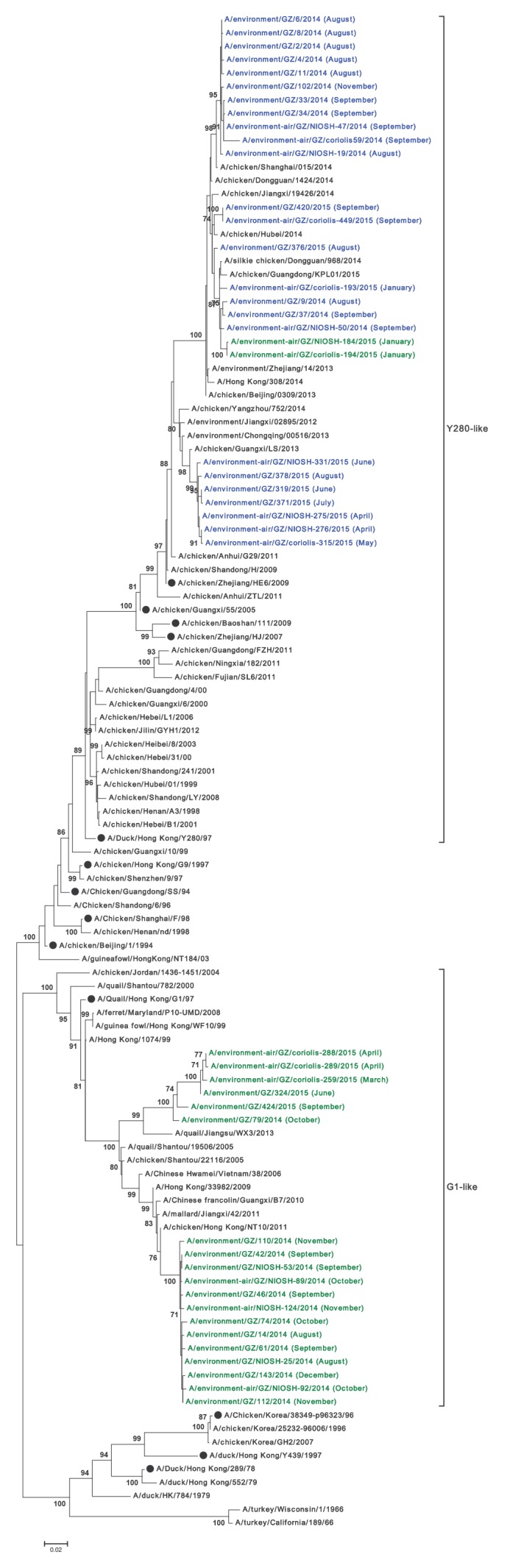
Phylogenetic analysis of the haemagglutinin gene of avian influenza A(H9N2) viruses isolated from a wholesale market and a mixed animal market in Guangzhou, China, July 2014–October 2015 (n=46)

HA: haemagglutinin; G1-like: A/quail/Hong Kong/G1/97-like H9N2 virus; Y280-like: A/Duck/Hong Kong/Y280/97-like H9N2 virus.

The H9N2 virus was the most frequently isolated subtype from the markets in Guangzhou we sampled, with a total of 58 isolates of H9, H9/H7, or H9/H5 subtypes (Table 2). We performed a phylogenetic analysis of the HA gene of 46 selected H9N2 viruses isolated from the wholesale market (10 air samples, 15 environmental swabs) and the mixed animal market (10 air samples, 11 environmental swabs) in Guangzhou city. The H9N2 viruses isolated from the air and environment from the same market were genetically related. Furthermore, the H9N2 viruses isolated from the wholesale and the mixed animal markets were separately clustered into two clades (Figure 3).

The coding sequence of the HA gene (1,093 nucleotides, 115–1,207 nucelotides from ATG) was aligned for the phylogenetic analysis. H9N2 viruses isolated from the wholesale market and mixed animal market in Guangzhou are shown in blue and green, respectively. The black circles indicate the representative strains of distinct H9 lineages and the vaccine strains.

The phylogenetic tree was constructed by maximum likelihood method with bootstrap analysis (n = 1,000) using MEGA (version 6.0) software.

The H9N2 viruses isolated in the wholesale market shared high nucleotide homology (93.5–100%) and all clustered with the A/chicken/Zhejiang/HJ/2007 virus (G57 genotype), which evolved from A/Duck/Hong Kong/Y280/1997 (Y280 genotype) and has become dominant among chickens in China since 2010 [16]. At the mixed animal market, where minor land poultry were sold, the majority of H9N2 isolates (17/19) clustered together with the the A/quail/Hong Kong/G1/1997 (G1-like) virus, with high nucleotide homology (91.4–99.9%), except for two isolates collected in January 2015 by the NIOSH and Coriolis air samplers, which were clustered with the G57 genotype. The G1-like H9N2 viruses have been commonly detected in China since the late 1990s from minor poultry species such as quails and chukar partridges [17,18]. 

## Discussion

Influenza viruses are transmitted via different but non-mutually exclusive modes [4]. Infections are mediated via virus-laden particles of various sizes that confer fomite, droplet or airborne transmission [19-21], but the modes of transmission for human zoonotic infections by avian influenza viruses at the human–poultry interface are not well defined. In our study, we determined the quantity, viability, subtype and size of influenza virus-laden particles in the air at three types of live poultry markets in Guangzhou city. Although our study is limited to a small number of markets in Guangzhou city and Hong Kong SAR and the results should be interpreted with caution, we show that viral RNA or viable avian influenza viruses of H5, H7 and H9 subtypes with human zoonotic infection potential are readily detectable in the air, suggesting the feasibility of airborne transmission of avian influenza viruses at the human–poultry interface. Furthermore, human activities, such as operation of de-feathering machines commonly used at live poultry markets in China, may facilitate generation of viable virus-laden particles in the air. In contrast, the negative air sampling results obtained at the wholesale market in Guangzhou on or after market closure day suggest that appropriate interventions may reduce the viral load effectively in the environment. While poultry markets are not common in Europe, the result is consistent with the detection of influenza viral RNA in the air at poultry farms sampled during avian influenza outbreaks in the Netherlands [22]. Our study provides experimental evidence showing that viable avian influenza viruses can be detected in the air where live poultry are kept, which is consistent with previous reports that detected viral RNA and infectious influenza viruses at swine barns or at live pig markets in the United States [23,24]. Although it is difficult to compare our results with those reported previously due to differences in the air samplers used, the concentrations of viral RNA we detected in the air at the live poultry markets were comparable with those detected at the swine barns in the United States in 2011 [24].

Our results suggest that poultry workers in the live poultry markets are constantly exposed to high viral loads in the air and the environment, but human symptomatic infections caused by avian influenza viruses in this population remain uncommon. Excluding the samples collected at the slaughtering area of the Guangzhou wholesale market (site A2) and from the live poultry market in Hong Kong, using the NIOSH bioaerosol sampler, viral RNA or viable virus was identified predominantly from particles > 4 µm (16 viable isolates of 58 samples collected), occasionally from 1–4 µm (1/58), and none from particles < 1 µm (0/58). Previous studies that analysed particle deposition suggest particles < 3 μm are more likely to deposit in the deep lungs [25] where avian influenza viruses with binding specificity for α2,3-linked sialic acids preferentially replicate [26]. In addition, seroepidemiological studies have reported a limited number of cases with low levels of neutralising antibody titres using hemagglutination inhibition assay or neutralisation assay [27-29]; however, the mechanism of cross-protection may be via non-neutralising antibodies or T-cell response. Further studies are needed to evaluate the percentage of subclinical infections and to assess the cross-protective adaptive immune response between poultry workers and the general population.

The H9N2 avian influenza virus ubiquitously present among land-based poultry in China and other countries [30] was the predominant subtype detected from the air and environmental samples in our study. Genetically diverse H9N2 viruses have been shown to possess human-like receptor binding specificity [31], transmission potential among ferrets [32] and have provided the internal genes for the H7N9 or H10N8 viruses that have caused fatal human infections since 2013 [33]. Unlike highly pathogenic viruses of H5 subtype that replicate systematically and cause high mortality, the low pathogenic H9N2 and H7N9 viruses generally do not cause apparent clinical signs in infected poultry [30,34]; this poses a challenge in identifying the infected birds for infection control and facilitates the spread of the H9N2 and H7N9 viruses in live poultry markets. H9N2 and H7N9 viruses are known to replicate more efficiently in the respiratory tract than the gastrointestinal tract of the land-based poultry [34,35], and the highly prevalent H9N2 virus has been the dominant subtype detected in the air at the poultry markets, as shown in the present study. Determining the viral loads and subtypes from oropharyngeal and cloacal swabs from different poultry species may help to understand the effect of viral respiratory tropism versus the quantity of virus-laden particles released in the air. We also observed segregation of species-adapted H9N2 lineages at different markets; further studies should investigate if the segregation is due to repeated re-introduction of a species-adapted virus as a result of selling different species at different markets or if insufficient cleaning of the environment facilitated the persistence and segregation of the H9N2 virus.

Among the three different types live poultry markets in Guangzhou, we noted higher virus isolation rates from air samples collected at the wholesale market and the mixed poultry market than that of the retail market, suggesting the number of poultry sold on site may affect the quantity of viable virus detected in the air. Cleaning practices, such as the market rest day, may have an impact as well. In addition, we noted a higher detection rate and isolation rate from particle > 4 μm, regardless of the viral subtype, suggesting that there is no correlation between avian influenza A subtype and virus detection at specific particle sizes. Since the subtypes detected in the air correlate well with the subtypes detected from the environment (water, faecal droppings and surfaces), the prevalence of a subtype in poultry (e.g. H9N2) may be a major contributing factor to the subtype detected in the air; however, other factors including viral tropism in poultry should also be considered. Temperature and relative humidity can affect viral viability and the sizes of virus-laden particles in the air. However, we did not observe a strong impact of temperature and humidity on viral detection at specific particle sizes; a longer observation period and/or frequent sampling will be needed to address this question.

Taken together, our results indicate the possibility of airborne transmission for avian influenza A viruses and may explain some human cases who appear to have acquired H7N9 infection by visiting live poultry markets but without direct or indirect contact to poultry [8]. Furthermore, the observation that known zoonotic infections have been in people with transient contact with, or passing the vicinity of live poultry markets – rather than those working within them, who are clearly exposed to avian influenza viruses on almost a daily basis – suggests a role for host susceptibility as one of the key determinants of zoonotic infection.
